# Potential contribution of alveolar epithelial type I cells to pulmonary fibrosis

**DOI:** 10.1042/BSR20171301

**Published:** 2017-11-21

**Authors:** Michael Kasper, Kathrin Barth

**Affiliations:** Medical Faculty ‘Carl Gustav Carus’, Institute of Anatomy, Technical University of Dresden, Fetscherstr. 74, Dresden D-01307, Germany

**Keywords:** alveolar epithelial type I cell, pulmonary fibrosis, P2X7R

## Abstract

Pulmonary fibrosis (PF) is characterized by inflammation and fibrosis of the interstitium and destruction of alveolar histoarchitecture ultimately leading to a fatal impairment of lung function. Different concepts describe either a dominant role of inflammatory pathways or a disturbed remodeling of resident cells of the lung parenchyma during fibrogenesis. Further, a combination of both the mechanisms has been postulated. The present review emphasizes the particular involvement of alveolar epithelial type I cells in all these processes, their contribution to innate immune/inflammatory functions and maintenance of proper alveolar barrier functions. Amongst the different inflammatory and repair events the purinergic receptor P2X7, an ATP-gated cationic channel that regulates not only apoptosis, necrosis, autophagy, and NLPR3 inflammosome activation, but also the turnover of diverse tight junction (TJ) and water channel proteins, seems to be essential for the stability of alveolar barrier integrity and for the interaction with protective factors during lung injury.

## Introduction

**Figure 1:**
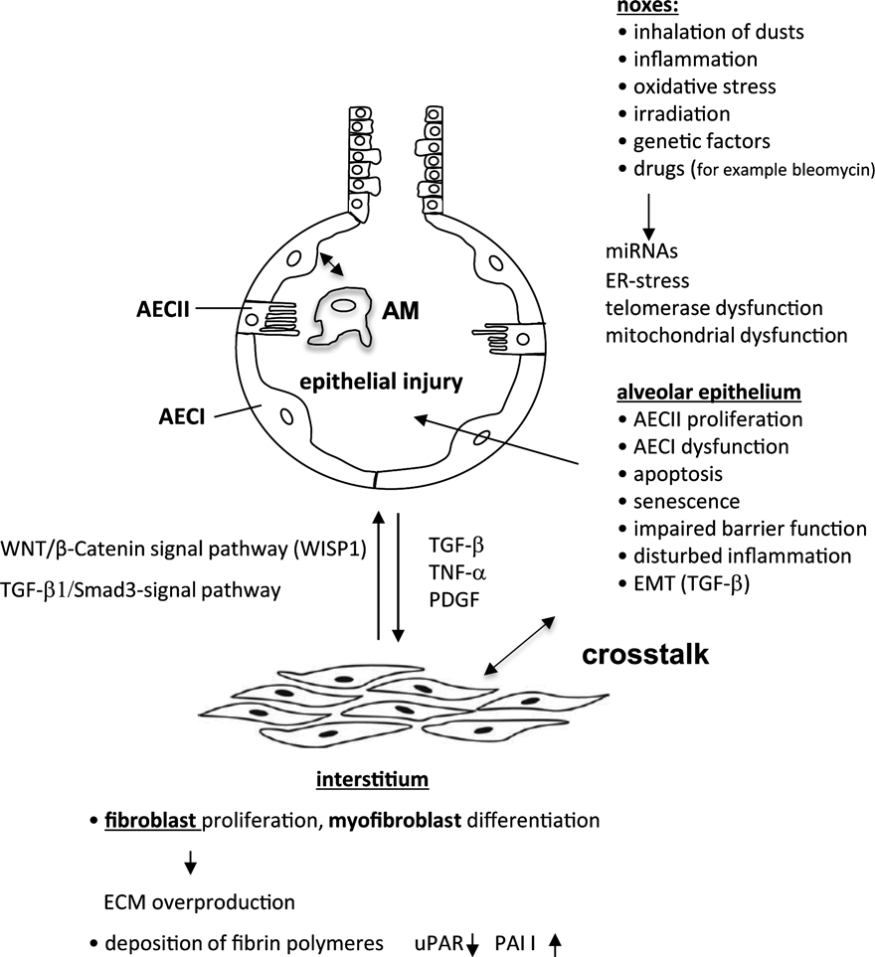
Involvement of AECs in PF

Pulmonary fibrosis (PF) represents a disorder of the lower respiratory tract, which is characterized by fibrosis and inflammation of the pulmonary interstitium, ultimately leading to destruction of alveolar architecture. The tissue injury may be caused by inhalation of dust and chemical agents, radiation, or by intratracheal or intravenous administration of drugs. The process of fibrogenesis is often compared with aberrant wound healing, involving incomplete tissue repair and remodeling. Interactions between inflammatory, fibroblastic, and epithelial cells appear to play a crucial role [1]. Idiopathic PF (IPF) belongs to a family of lung disorders known as interstitial lung diseases (ILDs). IPF is the most common form of PF in humans. The histopathological pattern that identifies patients with IPF is the usual interstitial pneumonia (UIP) [2]. IPF contributes to an estimated overal incidence of 7–10 cases per 100000 per year in the United States and in Europe [3,4] and has a progressive and fatal course. The etiology is unknown but genetic factors combined with environmental, epigenetic factors [5] play a role in this type of fibrosing interstitial pneumonia. Advanced age is a further important risk factor for IPF development [6]. Histologically, IPF is characterized by accumulation of fibroblasts, myofibroblasts, alveolar, and interstitial macrophages and by excessive extracellular matrix (ECM) deposition, leading to lung scarring and subsequently to chronic respiratory failure. Current evidence suggests that the fibrotic response involves abnormally activated alveolar epithelial cells (AECs) and that the injury starts with the damage of type I AECs (AECI), which cover the majority of the alveolar surface. When AECI are destroyed, AECII undergo hyperplastic proliferation to cover the denuded basement membrane and in an obvious chaotic fashion, apoptosis and differentiation of AECII into AECI occur for abberant replacement and repair of the alveolar surface [7–11] (Figure 1).

The exact pathomechanisms underpinning the initiation and advancement of PF are not clearly understood. Recent research including the relevant experimental findings from different animal models of PF suggests that in the lung, an imbalance of the wound repair process is regarded as the most important event during fibrogenesis (for excellent reviews, see [12–15]). Others stress the importance of balanced proinflammatory and anti-inflammatory mechanisms in PF [16,17]. There is, however, a great controversy over an initial role of inflammation in the development of PF [18,19].

AECs: the AECI represent more than a simple part of the air–blood barrier that comprises epithelium, endothelium, and their fused basement membranes. AECs regulate and control the fluid homeostasis in the alveolar wall and AECI, in particular, express diverse ion and water channels and tight junction (TJ) proteins [20–23]. In addition, their innate immune function probably contributes to the pathogenesis of PF [24]. AECs are the main site of production of platelet-derived growth factor (PDGF), transforming growth factor (TGF)-β, and tumor necrosis factor (TNF)-α, which are all central factors for the development of PF [25–27]. Also, endothelin (ET)-1, a multifunctional peptide able to induce mesenchymal cell mitosis is strongly up-regulated in AECII covering fibroblastic foci [28]. Connective tissue growth factor (CTGF), a chemotactic and mitogenic factor for fibroblasts, is also up-regulated in AECII and fibroblasts in IPF lungs [29]. All other structural cells of the lung such as alveolar macrophages (AMs), fibroblasts/myofibroblasts, mast cells, lymphocytes, endothelial cells, pericytes, lymphocytes as well as further epithelial cells including club cells and distal bronchial epithelial cells, form an inflammatory and repair network to maintain cellular homeostasis. The individual and specific role of each member of this regulatory system in the development and progression of PF is largely unknown. The aim of the present review is to summarize the main events of fibrogenesis with special focus on the contribution of the alveolar epithelium, mainly the AECI, to inflammatory/innate immune functions as well as to alveolar repair mechanisms, including the processes of senescence, apoptosis, and autophagy. In contrast with the more current view to emphasize the AECII cell dysfunction as a central event in fibrogenesis, we focus on AECI cell injury, describe some of the known AECI-selective proteins and discuss their involvement in disturbed barrier function in AECs leading to the complex pathological alterations in PF. Details of AECI cell biology have been summarized in excellent reviews [30,31] and are not described here. The role of epigenetic factors known for their contribution to fibrosis [32] and aspects of ageing and recapitulation of developmental pathways in epithelial remodeling [33–35] are also not included in this review.

## AECI cells under physiological conditions: its role in fluid homeostasis and proper barrier function

**Figure 2:**
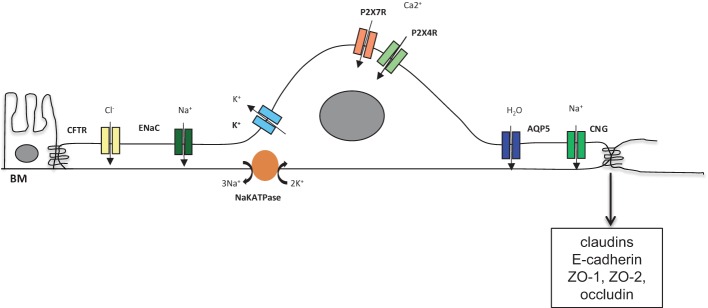
Possible AECI related ion channels and junctional proteins contributing to PF

For the exact description of the involvement of the alveolar epithelium in inflammatory and repair processes during remodeling processes in PF, it is necessary to distinguish between AECI and AECII biology. Two cell types populate the alveolar epithelium in normal adult lungs; AECI and AECII. AECI cover over 95% of the internal surface area of the lung. AECI are branched cells with multiple apical surfaces that extend into adjacent alveoli. The apical surface area of one AECI is very large in comparison with most cells (~5000 μm^3^ in humans) yet they are very thin (0.2 μm in depth) to facilitate efficient gas exchange. The basement membranes of AECI and capillary endothelial cells are fused to form the main barrier to gas exchange. AECI are important in the regulation of alveolar fluid balance and surfactant secretion by AECII in response to stretch [6–9]. AECII cover the remaining 2–5% of the lungs’ surface area. AECII are cuboidal cells situated between AECI. They contain characteristic lamellar bodies and apical microvilli. AECII have many known functions including the production, secretion, and re-uptake of pulmonary surfactant [36], regulation of alveolar fluid in normal lungs and during the resolution of pulmonary edema [37], and the synthesis and secretion of immunomodulatory proteins important for host defense such as surfactant proteins A and D [38]. AECI build an impermeable barrier to limit fluid infiltration into the alveolar airspace and to keep the alveoli relatively dry. The list of ion channels, transporters, and pumps at the surface of AECs is large. Diverse ion channels are identified, such as Na^+^- and K^+^-ATPase, amiloride-sensitive ENaC and the cystic fibrosis transmembrane conductance regulator (CTFR). CTFR is a cAMP-dependent Cl^−^ channel that regulates epithelial Cl^−^ and fluid secretion. In addition, AECI cell express cation-selective cyclic nucleotide-gated and K^+^ channels, GABA receptor and a ligand-gated chloride channel, and AQP5, a water channel [21,39]. The various channels and transporters enable the AECs to control transepithelial water flow. Airway epithelial ion transport mechanisms can be modulated by various signaling molecules; for example the ones initiated by activation of purinergic receptors [40,41]. In addition, AECI cell junctions are predominantly responsible for alveolar epithelial barrier function through the large surface area of the extremely flat AECI [42]. Repetitive tissue injury causes epithelial barrier dysfunction and results in host responses including interactions of various cells with soluble factors to restore normal lung structure and function. Very little data exist on the consequences following loss of this barrier function. Indeed, not much is published on the potential role of intercellular junctional complex proteins in maintenance of the epithelial barrier integrity and development of PF. The apical component of the junction complex is formed by TJs, which play a crucial role for the regulation of the transepithelial paracellular transport and which are also essential for innate immunity, proliferation, and cellular differentiation. The main components represent the membrane proteins claudin and occludin together with scaffold proteins known as zona occludens (ZO-1, ZO-2, and ZO-3) proteins [43]. The epithelial barrier function of TJs in the lungs is mainly established by claudins. Claudin-5 is expressed in pulmonary epithelial as well as in endothelial cells [44]. Claudins-4 and -18 are present in AECI–AECI juctions, whereas AECI–AECII junctions additionally contain claudin-3 [45,46]. Particularly claudin-18.1 is unique in the lung and the dominant transcript in AECI [47], which maintains alveolar fluid homeostasis. Junctional architecture between AECI is disrupted in the absence of claudin-18.1, where AECI–AECI junctions appeared ruffled and splayed as compared with the normal overlapping junctions observed in wild-type mice [48]. Increased paracellular permeability connected with the entry of antigens, toxins, and protein-rich fluid into alveolar spaces may be the result of deletion of TJs [8]. Since proinflammatory cytokines TGF-β, TNF-α, and IL-1β and reactive oxygen species (ROS) impair alveolar epithelial channel expression and function [49], similar effects can be expected under conditions of PF. Figure 2 summarizes some important channels and AECI-related junctional proteins involved in proper barrier function of the alveolar epithelium.

## AECI and tissue repair during fibrosis

Investigating AECI injury and repair is difficult because these AECI are not easily recognized by light microscopy. To date, most studies have relied on visualizing AECI, and in particular necrosis of AECI, by electron microscopy. However, a number of proteins have been identified which are expressed by AECI but not by AECII (reviewed in [50]). These proteins, and antibodies against them, can be used in a variety of assays to quantitate the extent of AECI necrosis and also investigate AECI repair. Unfortunately, most selective markers of AECI cells, for example T1α and receptor for advanced glycation end products (RAGE) are not functionally characterized [51]. Damaged AECI are replaced by AECII [52–55], reviewed by [56]). The studies by Evans et al., in particular, provide the foundation for our current knowledge of the response of the alveolar epithelium to injury [57].They examined the ultrastructural changes to the alveolar epithelium following exposure to the oxidant gas, nitrogen dioxide (NO_2_). In alveoli adjacent to terminal bronchioles, AECI were maximally damaged at 8–12 h [57]. By 48-h exposure, the denuded basement membranes were repopulated by cuboidal epithelial cells, some of which were AECII. Evans et al. [52] followed the fate of proliferating AECII using a combination of autoradiography and electron microscopic analysis. Proliferating cells were marked with tritiated thymidine, which is incorporated into newly synthesized DNA during the S-phase of the cell cycle. At 48 h of NO_2_ exposure, most marked cells were AECII, however by 96 h the number of marked AECI had dramatically increased while the proportion of marked AECI had decreased. In addition, over the ‘chase period’ there was a transient increase in markers associated with AECs with characteristics of both AECI and AECII (i.e. AECI-like cells but spread out over the basement membrane or AECI-like cells containing occasional microvilli and lamellar bodies) [52]. These studies demonstrate three important aspects of alveolar epithelial injury and repair (in response to NO_2_): first, that the loss of AECI can precede AECII cell proliferation. Second, that alveolar epithelial repair involves the transformation AECII (or a subset) into new AECI. Third, that AECII transforming to new AECI do so via definable structural intermediates.

More recent studies have refined the role of AECII in AECI repair and raised alternative mechanisms for AECI repair [58–61]. There may exist subpopulations of multipotent AECII capable of generating multiple differentiated cell types, including AECI [61]. Damaged AECI may also be replaced by bone marrow and non-ciliated bronchiolar epithelial progenitor cells [58,59]. It has been assumed for a long time that AECI were ‘terminally’ differentiated. However, experiments designed to investigate the role of mechanical load in fetal lung development suggest that AECI have the potential to transdifferentiate to AECII [60]. We do not know whether AECI are capable of replacing damaged AECII in injured lungs. In areas with pathomorphological signs of PF *in situ*, ‘intermediate’ cells showing properties of AECI and AECII exist [33,62]. The phenomenon of ‘intermediate’ cells in PF expressing AECI and AECII cell markers together [63,64] and the presence of subpopulations of intermediate or transitional AECs in the normal adult lung [65] need further evaluation. AECI cells can obviously no longer be regarded as ‘terminally’ differentiated, but are able to perform a conversion into AECII cells [66].

Further, under experimental and *in vitro* conditions it is often not sufficiently clarified, which cell types were used. Freshly isolated AECII often express AECI-specific genes [67,68]. Importantly, immortal alveolar epithelial lines do not unequivocally exhibit the phenotype of one or the other AEC type, and in many cases isolated AECI showing fibroblastic/mesenchymal transformation with expression of α-SMA and vimentin [69]. In the last years, many investigators have provided strong evidence for epithelial–mesenchymal transition (EMT) *in vitro* employing primary and immortal AECII-like cells, particularly in response to TGF-β. We do not know how EMT of AECs contributes to PF in a meaningful way, whether AECI and/or AECII undergo EMT, and whether AECI-related proteins are involved [62]. Careful immunohistological evaluation of human IPF samples and of tissues from a bleomycin (BLM)-induced mouse model employing AECI and AECII-specific markers failed to detect any coexpression with mesenchymal markers [62]. In another study, inhibition of plasminogen activator inhibitor-1 (PAI-1) activity blocked the TGFβ-dependent EMT and limited the development of BLM-induced PF in mice [70]. It remains open, whether this effect can be specifically addressed to AECI.

Despite the following processes of proliferation, apoptosis, senescence, and autophagy are not exclusively related to the AECs. The same processes occur in non-epithelial cells. There is no doubt on a predominant role of these events in the epithelial compartment of the lung [6].

After the loss of integrity of the alveolar epithelium in PF, together with the disruption of basement membrane integrity and the collapse of the alveolar structure, alveolar repair starts with the development of hypertrophy and hyperplasia of AECII, whereas the number of AECI is reduced. AECII proliferation results in abnormal re-epithelialization over the course of several days. This process is seriously impaired in PF [71] leading to cuboidal metaplasia and alveolar bronchiolization [72]. For the role of the other epithelial cell types in the distal bronchial epithelium of the lung such as secretory club and goblet cells, ciliated, basal, and neuroendocrine cells and their contribution in this process via the secretion of anti-inflammatory factors see a recent review [71]. Since this review focusses on the specific role of AECI in lung cell homeostasis, processes of apoptosis, cellular senescence, and autophagy have to be discussed.

### Apoptosis

There is growing evidence that apoptosis of AECII is a major factor in IPF. Moreover, it might be the initial damaging event in the development of PF [73]. Two pathways of programmed cell death: extrinsic and intrinsic, exist. The extrinsic pathway involves the extracellular ligands Fas/CD95, assembly of a death-inducing signaling complex and activation of caspase-8 followed by the activation of effector caspases-3 and -7. The intrinsic pathway involves the activation of the proapoptotic Bcl-2 family members, the cytochrome *c* release, formation of the apoptosome complex, activation of caspase-9, and finally caspase-3 and -7. Preferably the fas/fas ligand pathway but also the intrinsic pathway participate in PF [74,75]. Most apoptosis has been seen in AECII adjacent to underlying myofibroblasts [76,77]. Remarkably, TGF-β1 was shown to enhance the fas-mediated epithelial cell apoptosis via caspase-3 activation [78]. Other reasons for the apoptosis of AECs are endoplasmic reticulum (ER) stress after mutation of surfactant protein C (SP-C) [79], oxidative stress, and angiotensin 2 [80], for review see also [19]. Epithelial apoptosis is accompanied by damage to the basement membrane leading to the release of growth factors and chemokines by neighboring inflammatory cells in the alveolar wall. It was shown that many of the products of epithelial cell injury may stimulate myofibroblasts to produce ECM components, most notably collagen. What about AECI? To the best of our knowledge no convincing data exist that in contrast with AECII, AECI undergoes apoptosis. Ultrastructural data describe necrotic cells after injury [81–83]. The extreme sensitivity of AECI cells to injury may be caused by the limited number of mitochondria and the flatness of the cells.

### Senescence

This is a process of cellular ageing caused by telomere shortening and characterized as irreversible growth arrest, hypercellularity, expression of cyclin-dependent kinase inhibitor (CDKI), and senescence-associated secretory phenotype (SASP) [84]. Typical markers of senescence by using immunohistochemistry are p16, p21, and senescence-associated β-galactosidase (SA-βgal). Cellular senescence has been implicated in the pathogenesis of IPF [6] and seems to be regulated via miRNAs of the *miR-34* family [85,86].

Telomerase-deficient mice or mice with Trf1 deletion in AECIIs develop PF [87]. IPF patients exhibit telomere shortening in peripheral blood cells, but also in the AECs [88]. In active lesions of IPF lungs, p21 was up-regulated and cell cycle regulatory proteins Cyclin D1 and SOCS3 were significantly enhanced [89]. Waisberg and collegues [90] have shown that AECII with low telomerase expression and high apoptosis are found in unaffected areas of lung tissues from patients wih IPF. Persistent DNA damage and SASP were found in BLM-induced fibrosis models [91]. Minagawa et al. demonstrated accelerated senescence of AECII in active fibrosing lesions [92]. Telomere dysfunction of AECII induced by conditional deletion of the shelterin component telomeric repeat binding factor 2 implicates altered immune functions resulting in up-regulation of many inflammatory cytokines [93]. Data about senescence in AECI are completely missing with one exception: a possible link comes from the p53–uPA fibrinolytic system involving both types of AECs [86]. PAI-1-deficient mice is more or less resistant to BLM and other kinds of lung injury [94]. PAI-1, is present in AECI [95] and AECII as well [96]. PAI-1 is increased in senescent cells and regulates the cell cycle and apoptosis involving pulmonary SP-C [94], caveolin-1, and intercellular adhesion molecule (ICAM-1) [94].

### Autophagy

**Figure 3:**
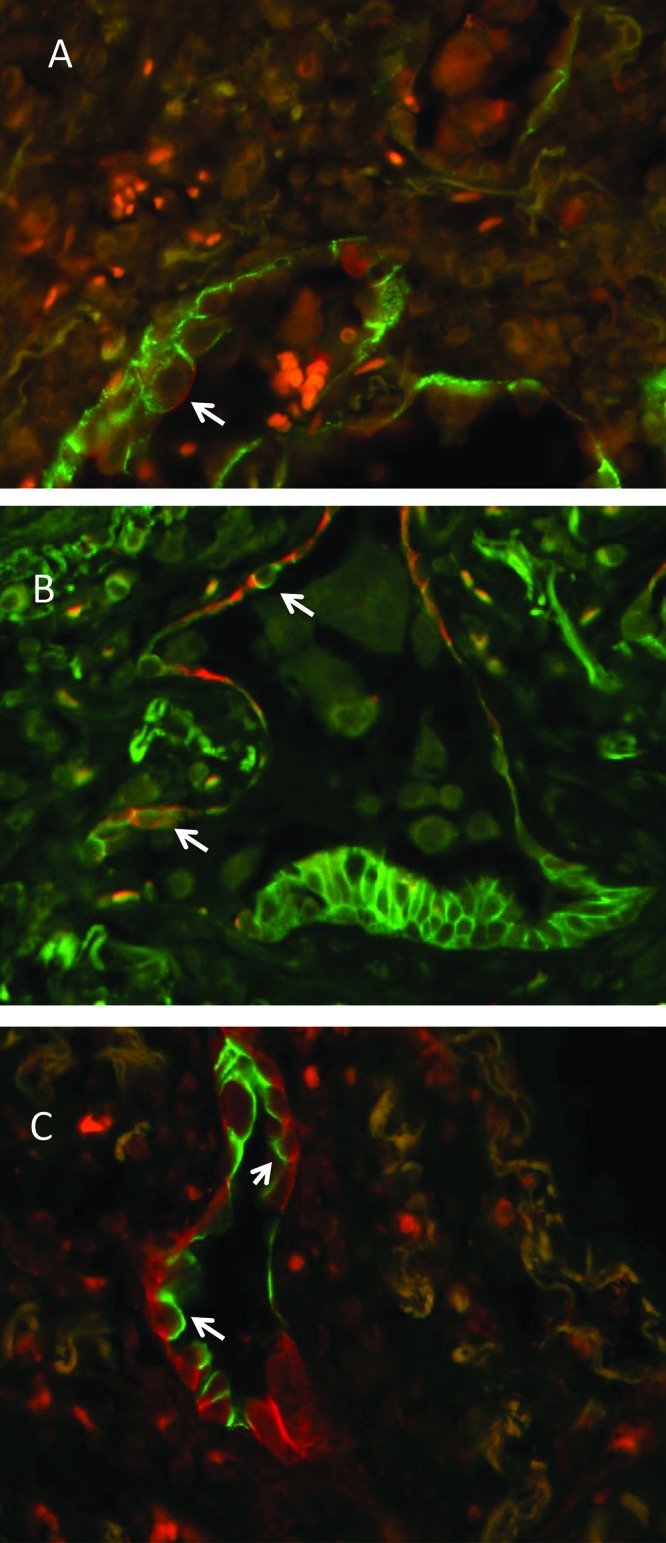
Presence of “intermediate” AECs in IPF lung samples Immunohistochemical evidence of intermediate AECs in human IPF analyzed within a frame of a previous study [62]: co-localization of CD44v9, AECII specific (FITC) and *Lycopersicon esculentum* agglutinin, AECI specific (Texas Red) (**A**), E-cadherin, AECII specific (FITC) and RAGE, AECI specific (Texas Red) (**B**), and of E-cadherin, AECII specific (FITC) and ICAM-1, AECI specific (Texas Red) (**C**) in AECs of intermediate type. Arrows indicate double labeled cells.

Close to cellular senescence is an autophagy of AECs. Autophagy describes a cytoprotective mechanism of lysosomal self-degradation of cells following cellular stress caused by ER stress, oxidative stress, hypoxia, immune cell activation and exposure to different exogenous noxes such as bacteria and airway pollutants. As a consequene, autophagy-related gene (ATG) activation leads to the induction of many inflammatory pathways. It has been shown that diminished autophagy with insufficient degradation of damaged cellular organelles, intracellular microbes, and long-lived proteins contribute to cellular senescence and accelerated ageing. There is evidence that autophagy plays important role in pulmonary diseases [6]. A very good example is the cigarette smoke induced mitophagy in chronic obstructive pulmonary disease (COPD). Insufficient mitophagy activation is associated with excessive mitochondrial ROS production leading either to cell senescence or apoptosis [6]. Genes involved in autophagy are beclin-1, ATG-5, lysosome-associated membrane protein (LAMP)2A, light chain 3 isoform B (LC3B), and others. The autophagic protein LC3B has been shown to be involved in transdifferention of AECII into AECI under hyperoxia conditions [97]. Autophagic activity and ATG4B expression, the main ATG4 protease for autophagy in mammalian cells, increase during experimental BLM-induced PF [98]. Immunohistochemistry for ATG4B revealed immunopositive AECII, but not AECI cells in BLM-exposed mouse lung tissue. Human samples from IPF patients exhibited a positive staining of hyperplastic and hypertrophic AECII overlying the fibroblastic foci, of bronchial epithelial cells and a few interstitial cells [98]. ATGB4 knockout mice exhibited increased apoptosis of AECII cells and more extensive and severe fibrosis [98], suggesting a protective role of this protease and autophagy in PF. Inflammatory mediators released after ATG activation are IL-6, IL-8, IL-1A and -B, and TNF-α [99], as well as IL1-2B and CXCL1/KC [98]. In PF, insufficient autophagy has been hypothesized to be associated with cellular senescence, which results in ER stress of AECII and myofibroblasts [6]. The same group demonstrated selective immunoreactivity for p21 and SA-βgal in AECs covering fibrotic foci in IPF lungs, but not in myofibroblasts [100]. We have some evidence that AECs of the intermediate type may be senescent cells, since intermediate cell are very often found in fibrotic foci and in areas of alveolar bronchiolization (Figure 3).

The presence of intermediate cells in active areas of PF remains obscure. The same can be observed in other fibrosis samples in BLM- and radiation-induced experimental models [33,63,101]. The evidence that senescence and autophagy of cells is linked with disturbed transdifferentiation of AECII into AECI has not yet been proved in PF. Markers of autophagy and senescence still have to be combined with AECI and II markers in immunohistochemical co-localization studies in IPF lungs. The AECII–AECI transition during fetal development of normal lung is dependent on fine-tuned Wnt/β-catenin signaling [102]. Altered Wnt pathways are accompanying alveolar epithelial remodeling processes in lung cancer and in PF [103]. Several Wnt ligands such as Wnt7A and Wnt3A have been shown to be able to induce cellular senescence in lung disease (reviewed in [104]). Given the importance of autophagy in cellular homeostasis during fibrogenesis, it is also important to study the purinergic P2X7 receptor (P2X7R), a new modulator of metabolic oxidative stress mediated autophagy in diverse disease pathologies [105]. P2X7 knockout mice show decreased fibrosis and inflammation in an experimental nonalcoholic steatohepatitis model [105]. This model exhibits increased metabolic oxidative stress followed by the expression of ATGs like LAMP2A and heat-shock cognate 70. Deletion of the *P2X7R* gene protects the cells from injury. One of the possible mechanisms is a Ca^2+^-dependent lysosomal alkalization after stimulation of the P2X7R with ATP. The increased lysosomal pH is followed by a higher lipid oxidation leading to an impairment of the degradation of autophagic vesicles [106]. In intestinal epithelial cells activation of P2X7R induces apoptosis and autophagy [107]. An important, yet undefined role of P2X7R for lung pathologies has to be discussed, since this receptor is selectively expressed in alveolar macrophages and in AECI (see below) [108–110].

## Putative signaling pathways connected with AECI cells

### Toll-like receptors

TLRs as part of the innate immune system belong to a group of receptors termed pattern recognition receptors (PRRs), and trigger specific responses that promote the repair and restoration of tissue function, including inflammation and wound healing. They recognize specific molecular patterns (PAMPS) that are present in bacteria, and viruses, including lipid-based bacterial cell wall components such as lipopolysaccharide (LPS) and lipopeptides, microbial protein components and nucleic acids such as single-stranded or double-stranded RNA. They also react to certain danger-associated molecular patterns (DAMPs) that are endogenous molecules released from necrotic or dying cells and the environment (reviewed in [111]). DAMPS are signature molecules that include nucleic acids, ECM fragments, cytoskeleton components, small molecules like uric acid and ATP, as well as large proteins such as heat shock proteins (HSPs), S100 proteins or high mobility group box protein 1 (HMGB1) [112]. DAMPs activate the immune system through interactions with TLR2 or TLR4. Others, such as ATP and its receptor P2X7R, co- operate with other inflammatory stimuli to induce activation of the inflammasome (see below). Activation of TLRs by PAMPS and DAMPS is leading to the initiation of various inflammatory pathways characteristic for an adaptive immune response. In AECII, TLRs 2 and 4 regulate innate immune response to different endogenous and exogenous ligands such as LPS, hyaluronic acid, and others. The contribution of TLRs to the development of PF and wound healing is not well understood [112]. There is evidence for a protective role of TLR4 in BLM and silica-induced fibrosis [113,114]. Double knockout of TLR2/4 mice are not protected from the development of fibrosis in a BLM model [115] and exposure with radiation increased fibrotic response [116]. There is a controversy about the presence of TLR2 and 4 in AECI [117,118]. Increased number of AECII with increased immunoreactivity for TLR2 and 4 has been demonstrated in lung tissue of IPF patients [119].

### RAGE/HMGB1 axis

HMGB1 has been suggested to be another endogenous TLR ligand that contributes to inflammation in various models of injury via signaling through TLR2, TLR4, and the RAGE on inflammatory cells and on AECs [112]. HMGB1 is a ubiquitously expressed DNA-binding protein that stabilizes nucleosome formation, facilitates gene transcription, and amplifies an inflammatory response by stimulating the release of various proinflammatory cytokines [120].

HMGB1 is up-regulated in experimental PF and binds RAGE with high affinity [121,122]. RAGE is a member of the Ig super family of cell surface receptors and as member of the PPRs a key regulator of inflammation [123]. RAGE is a strong activator of the proinflammatory transcription factor NF-κB (nuclear factor-κB) and thereby represents an important signaling molecule in the innate immune system. In the lung, RAGE is selectively present in AECI, in some endothelial cells of larger blood vessels, and in alveolar macrophages [124–126]. Immunoelectron microscopic studies revealed the presence of RAGE at the basolateral aspect of AECI of mouse, rat, and human lung [124,127] opposite to the common basement membrane of the air–blood barrier. Loss of RAGE from AEC surface has been found in several experimental fibrosis models [124,128–132]. RAGE knockouts develop spontaneous fibrosis with age and paradoxically are resistant to BLM [133]. The opposite finding that the absence of RAGE worsened the fibrosis after BLM exposure, was reported by Ding et al. [134]. Recent data suggest that dysregulation of the ezrin/radixin/moesin (ERM)–RAGE complex might be an important step in rearrangement of the actin cytoskeleton during proinflammatory cytokine-induced EMT of human AECs [135]. Soluble RAGE (sRAGE), a decoy receptor in the extracellular space, is lost in many cases of lung injury and seems to be a marker of tissue damage and damage to the ECM. Administration of sRAGE decreases inflammation under various experimental conditions [136]. Another interesting still unexplored contribution of AECI to the inflammatory network may come from the known co-operation of ICAM-1 with RAGE in pulmonary endothelial cells to mediate leukocyte recruitment after LPS-mediated lung injury [137]. Since AECI selectively expresses both ICAM-1 [138] and RAGE, interactions of both inflammatory proteins are possible and should come into the experimental focus of AECs. Finally, RAGE and P2X7R represent two plasma membrane receptors that are able to bind DAMPs released by necrotic cells. Very recently, it was shown that RAGE and P2X7R are overexpressed after hypoxic stress. Both receptors, once activated by HMGB1 or BzATP, initiate a signaling pathway involving phosphorylation of Akt and Erk1/2 and nuclear translocation of NF-κB [139]. Hypoxia is one of the important causes of EMT in PF [140,141]. Therefore, AECI, which undergo necrosis in the early stage of fibrogenesis, are preferable candidates to realize similar mechanisms in the alveolar epithelium during fibrogenesis. Table 1 lists some important AECI-specific or -selective proteins under normal conditions not detectable in AECII, with putative functions in PF.

**Table 1:** Examples of AECI-associated proteins and properties related to PF

Protein	Characterization	Involvement in PF
PAI-1 [95]	Serin protease inhibitor	Coagulation disturbance, intra-alveolar coagulation, impairs alveolar epithelial repair [11,142], inreased PAI-1 in BAL fluid from IPF patients [143].
ICAM-1	Leukocyte adhesion, alveolar macrophage interaction	Marker of AECI injury [144]
Caveolin-1, -2	Structural protein of caveolae, plasmalemmnal microdomains (specialized lipid rafts), involved in selected signaling pathways	Involved in epithelial apoptosis and senescence, regulatory role for TGF-β [145]
RAGE	PPR	Modulation of fibrotic response, activator of proinflammatory transcription factors
P2X7R	Purinergic receptor	Initiates K^+^ efflux leading to the activation of the NLRP3 inflammasome, autophagy
AQP5	Water channel	Inflammatory signal potentiator [146]
T1α/podoplanin [147]	Transmembranous glycoprotein of unknown function, platelet aggregation	Marker of AECI injury [50], elevated levels in bronchoalveolar lavage of injured lungs, loss from alveolar lining layer in PF [148]

Abbreviation: NLRP3, Nod-like receptor pyrin containing 3.

### Caveolin-1 and -2 related pathways

Caveolae are flask-shaped plasmalemmal invaginations present in most mammalian cell types with a diameter of 50–100 nm. Many cellular functions have been addressed to caveolae of lung epithelial cells: endocytosis (entry of pathogens), transcytosis, calcium signaling, lipid metabolism, and signal transduction in cellular proliferation, apoptosis, senescence, and autophagy [98,149–152]. Caveolin-1 is a scaffold protein of caveolae, which are particularly abundant in AECI, but not in AECII of lung tissue. Caveolin-1 expression is necessary for the stable expression and membrane localization of caveolin-2. Caveolin-2 alone is insufficient to induce caveolae biogenesis [153]. Caveolin-1 directly interacts with signaling molecules and effects diverse signaling pathways regulating cell proliferation, apoptosis, differentiation, and growth. Thus, caveolin-1 can influence TGF-β1 signaling by direct inhibition of the receptor, inhibition of the receptor gene expression, termination of TGF-β signaling by endocytosis, and possibly affecting the activation of latent TGF-β [154].

The first evidence for the involvement of caveolin-1 in lung disease was provided by a study of radiation-induced fibrosis in rat and mini-pig lungs. This study revealed a dramatic loss of caveolin immunoreactivity in AECI [155]. The alveolar epithelial loss of caveolin-1 has been later shown in BLM-induced fibrosis models [156,157]. Mice deficient in caveolin-1 develop a lung pathology that resembles a fibrosis-like phenotype [158,159], but is paradoxically resistant to BLM [151].

Since caveolin-1 knockout mice contain no caveolin-2 protein in the lung [159] and caveolin-2 knockout mice develop similar pulmonary alterations [153], a specific pathogenetic role of caveolin-2 has to be taken into account. Interestingly, caveolin-2 knockout mice is more sensitive to BLM-induced injury and is not associated with alterations in the TGF-β-signaling pathway [160].

A linkage between the caveolin-1 signaling and cell adhesion molecules in the pathobiology of lung diseases was also reported [161]. Under experimental conditions of hypoxia, alveolar barrier function was impaired, e.g. expression levels of caveolin-1, claudins, occludin, and ZO-1 decreased. Transfection of AECII with a cDNA encoding for caveolin-1 for its up-regulation antagonized the hyperoxia-induced damage of alveolar epithelial barrier and TJ protein loss. Cell type specific effects may influence this interaction, since co-immunoprecipitation studies in MDCK II cells revealed a selective interaction of caveolin-1 with claudin-2 and occludin, but not with claudin-4 and ZO-1. Cholesterol depletion did not influence this interaction, thus indicating a lipid raft independent co-localization [162]. Caveolin-1 controls airway epithelial barrier functions as was demonstrated in the bronchial epithelium of asthmatic lungs [163].

**Figure 4:**
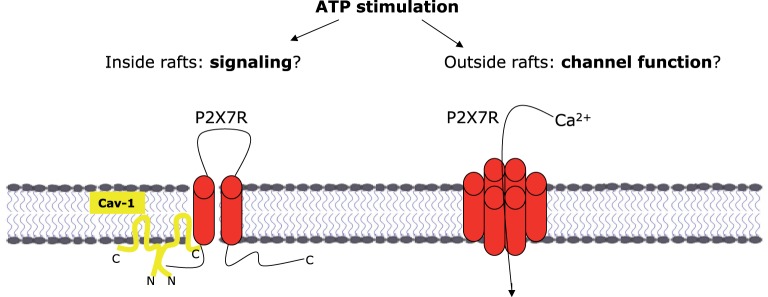
Dual function of P2X7R as ion channel or signal molecule

It was also shown that the caveolin-1 scaffold domain (CSD) binds multiple signaling molecules such as kinases and phosphatases to associate with channel complexes and thereby regulates Ca^2+^ entry in endothelial cells [164]. Corresponding data about the interaction of channel proteins with caveolin-1 in AECs are missing so far, with one exception: P2X4R and P2X7R are abundantly expressed in AECs, and are thought to play a role in regulating fluid hemostasis. There is evidence that P2X receptors are present in both raft and non-raft compartments of the plasma membrane and thus exhibit variable ATP sensitivity and also different functions [165,166] (Figure 4).

The interaction between caveolin-1 and both receptors has been characterized in the mouse AEC line E10 [166]. Using the biotinylation assay, it was found that only glycosylated P2X4R is exposed at the cell surface. Triton X-100 solubility experiments and sucrose gradient centrifugation revealed that P2X4R and P2X7R were partially localized in caveolin-1-rich membrane fractions. Suppression of caveolin-1 protein expression using shRNAs resulted in a large reduction in P2X4R and P2X7R levels. Using the GST pull-down assay, it was shown that caveolin-1 interacts *in vitro* with both receptors. Co-immunoprecipitation experiments confirmed the interaction between P2X7R and caveolin-1. Further, a marked reduction in P2X7R immunoreactivity was observed in lung sections prepared from caveolin-1-knockout mice, indicating that caveolin-1 expression was required for full expression of P2X7R protein [109,166].

## Effects of P2X7R in AECI and PF

The P2X7 purinergic receptor is a ligand-gated ion channel activated by extracellular ATP.

With the exception of C-terminus elongated by 120 AS, the structure of the P2X7R is similar to the other P2X receptors [167]. Within the extended C-terminal region exists a third hydrophobic domain, which can form a large, non-selective channel pore [168]. When ATP is applied at low concentrations (10 μM), the P2X7R acts as a ligand-activated channel and allows the passage of mono- and divalent cations (Ca^2+^, Na^+^, K^+^) through the plasma membrane [169,170]. Longer (10–30 s) or repeated applications of high ATP concentrations (>100 μM) lead to formation of a large non-selective pore in some P2X7R expressing cells, through which ions and low molecular weight metabolites with a molecular weight of up to 900 Da can flow [171,172]. As a result of this pore formation, necrosis or apoptosis occurs in most cases [173]. High concentrations of extracellular ATP are detected under pathological conditions such as injury, tissue space, inflammatory processes, and hypoxia. ATP then exits the cell and can activate the receptor and by this initiate the inflammation. Epithelial cells of the airways usually release ATP and other nucleotides in response to cell swelling, shear and compressive stress, and other physiological stimuli via conductive and vesicular pathways [174]. It is known that ATP can leave the cell via connexin hemichannels, volume-sensitive chloride channels, or dilated P2X7 receptors [175–178]. Paracrine effects of extracellular ATP on AECII and effects of metabolism of extracellular ATP for activation of innate immune response during lung injury are reviewed in [179].

In an experimental BLM-induced lung injury model, a reduced expression of claudins, especially claudin-18, along with lower levels of mRNA encoding TJ proteins was observed. Claudin-4 and claudin-18 are regulated in opposite direction in ALI, claudin-4 is increased and claudin-18 decreased [180,181]. However, it was also shown that in P2X7R knockout mice claudin-18 is increased and claudin-4 slightly decreased. In general, P2X7R knockouts seem to be more resistant to injury in lung disease models [108,182,183].

The expression of claudin-5 and claudin-18 decreased after 14 days of BLM injury, indicating that TJs of endothelial cells and AECs were severely affected in a BLM-induced injury model [180].

In airway epithelia, TNF-α also plays a major role in perturbing TJs. This protein acts via NF-κB, a major regulator of tissue inflammation [184]. TNF-α down-regulates paracellular epithelial barrier function in airway epithelia [185]. It negatively regulates TJ proteins which was revealed by genetic modulation of TNF-α in mice. Particularly claudin-2, -4, -5 and ZO-1 were affected in the lung, resulting in increased alveolar permeability [186]. Our former studies indicate an involvement of P2X7R and AQP5 in alveolar barrier function as well [187]. AQP5, the main aquaporin in peripheral lung, was down-regulated in 2–3 months old P2X7R knockout animals [187]. It was also shown that fibrotic areas were associated with decreased protein and mRNA expression levels of AQP5. It was further shown that AQP5 null mice as well as isolated epithelial cells deficient in AQP5 express enhanced barrier function. In addition, AQP5-mediated regulation of microtubule dynamics decreases paracellular permeability [188]. Furthermore, we have detected an involvement of AQP5 in the regulation of claudin-18 in AECs. Interestingly, claudin-18 is enhanced expressed in AQP5 knockout animals (unpublished data).

Claudin-4 and claudin-18 deficient mice exhibit phenotypes beyond a simple deficiency in barrier function. In both cases alveolar epithelial gene expression is significantly changed and proinflammatory responses are increased making the lungs more susceptible to injury. It is still unknown whether increased inflammation is directly induced by changes in barrier function or whether it is an independent process related to general changes in the control of lung cell phenotype caused by deficiency of claudin-4 or claudin-18.

### P2X7R and the inflammosome

In addition to alveolar macrophages and dendritic cells, epithelial cells of the lung may also express the Nod-like receptor pyrin containing 3 (NLRP3) inflammosome and IL-1β, a cytokine with major roles in inflammation in response to several stimuli release [189,190]. The NLRP3 inflammasome is well characterized and might be involved in the pathogenesis of ILDs, including IPF, asbestosis, and silicosis. The mechanisms of activation of inflammasomes are still not completely understood. The regulation of IL-1β production is a complex process starting first with the synthesis of pro-IL-1β and NLRP3 followed by inflammasome oligomerization, caspase-1 autoactivation, caspase-1-dependent cleavage of pro-IL-1β, and ending finally with the release of the biologically active, mature IL-1β. A number of endogenous and exogenous agents induce NLRP3 activation, classified as either PAMPs or DAMPs. Environmental pollutants including silica and asbestos induce formation of DAMPs [191].

NLPR3 knockout mice used in mouse models of asbestosis and silicosis exhibited decreased number of inflammatory cells in the lungs and lower cytokine production upon exposure to asbestos or silica, compared with control wild-type mice [192]. Remarkably, NLRP3 knockout mice showed less collagen deposition than wild-type mice 3 months after silica exposition.

EMT produces vimentin-positive myofibroblasts derived from the alveolar epithelium [193]. The type III intermediary filament vimentin is important for innate immune reaction and inflammation leading to acute lung injury (ALI) and fibrosis [194]. Vimentin is required for IL-1β maturation through its interaction with the inflammasome. The vimentin deficiency resulted in a blunted inflammatory response in the early stage of injury.

The inflammasome is further activated by the endogenous factor ATP [195]. An important protein in this pathway is the ATP-stimulated P2X7R, which is known to regulate the activation of the NLRP3 inflammasome. The P2X4 receptor, the second AECI-specific purinergic receptor, is also involved in the activation of the NLRP3 inflammasome [196].

A rapid increase in the extracellular ATP concentration, for example after damage of a tissue or after cell death, causes as an endogenous signal of danger and activates the NLRP3 inflammasome by the binding ATP to P2X7R which functions as ligand-controlled ion channel [197]. The effect of ATP may be transmitted by P2X7R, which causes the formation of pannexin-1 pores. Their opening is leading to a fast outflow of K^+^-ions from the cytosol [198]. The decrease in the cytoplasmic K^+^ concentration causes assembly and activation of the inflammasome leading to an autocatalytic cleavage of the inactive procaspase-1 to its active form. The inflammasome transmitted caspase-1-dependent proteolytic cleavage of inactive proforms of cytokines of the IL-1-family (pro-IL-1β, pro-IL-18) results in biological active forms, which the cell releases as a part of the inflammatory reaction. P2X7R knockout mice exhibited dramatically reduced lung inflammation and fibrosis, underlining the role of P2X7R in this disease [108]. Aside from the production of IL-1β, the inflammasome may also contribute to the improvement of cell survival by activation of protective mechanisms in ALI [183].

## Outlook

In the present review, we highlighted some aspects of the involvement of AECI in signaling mechanisms during repair processes and maintenance of alveolar barrier integrity in PF. Most of the specifications about the outstanding role of AECI during fibrogenesis remain unclear. In the past, AECI have received little attention compared with its neighbor in the alveolus, the AECII. The understanding of specific properties of AECI, in our opinion, is important for the understanding of their role in the initiation of PF. This review particularly summarizes some recent findings about the contribution of P2X7R in AECI to the pathogenesis of PF, which exert different not yet evaluated regulatory functions, and which offer potentially new approaches for fine-tuned therapeutic intervention in PF. One promising tool may be the blockade of the P2X7R. The first animal experiments have shown that a specific inhibitor of P2X7R, A438079, prevents the development of liver injury and fibrosis in a mouse model of liver fibrosis [199]. The levels of proinflammatory cytokines TNF-α and IL-1β and the activity level of NF-κB were significantly reduced via treatment with A438079. The first commercially available P2X7R antagonist are available and used in preclinical phase I and II trials of patients with inflammatory lung disease [200]. More broader clinical studies with P2X7R antagonist exist for painkilling and treatment of cancer and other inflammatory and autoimmune dieseases (reviewed in [201]).

In addition, drugs and gene therapies with properties to promote the alveolar barrier function and antibody therapies targetting AEC-derived growth factors offer exciting new possibilities to modulate the disease process. For example, anti-TNF-α antibodies prevent nitrogen mustard induced pulmonary injury and fibrosis in rat lung [202]. New delivery systems for antifibrotic drugs based on the incorporation into liposomes or loading on nanoparticles to pass the AECs are in progress [203,204].

Some other aspects of AECI biology require further evaluation:

- What about a putative functional heterogeneity of AECs and the cross-talk between AECI and AECII in the normal and injured lung? The interplay between the different signaling pathways regulating the channel and cell contact functions (including the gap junctional intercellular communication) to maintain a stable alveolar epithelial barrier integrity has to be explored and addressed in the different AEC subpopulations.
- The complex glycoconjugate pattern of the apical surface of AECI compared with AECIIs as found using lectin histochemistry [63,205] implicates the presence of diverse unknown receptor molecules on the AECI surface. Further, the presence of selective AECI markers such as P2X7R, T1α, epithelial ICAM-1, PAI-1 or RAGE, which are functionally not yet characterized, emphasizes a specific role of AECI.

Finally, to detect the diverse functions of AECs, we have to keep in mind that the AECs orchestrate diverse epithelial–macrophage, epithelial–leukocyte interactions in response to infectious and other noxes. Their cross-talk during the development of PF is a further largely unexplored chapter of lung research.

## Abbreviations

AEC: alveolar epithelial cell
AECI: type I AEC
AQP5: aquaporin 5
BLM: bleomycin
CTFR: cystic fibrosis transmembrane conductance regulator
ECM: extracellular matrix
EMT: epithelial–mesenchymal transition
HMGB1: high mobility group box protein 1
ICAM-1: intercellular adhesion molecule
IPF: idiopathic pulmonary fibrosis
LAMP: lysosome-associated membrane protein
LPS: lipopolysaccharide
NF-κB: nuclear factor-κB
NLRP3: Nod-like receptor pyrin containing 3
PAI-1: plasminogen activator inhibitor-1
PF: pulmonary fibrosis
P2X7R: purinergic P2X7 receptor
RAGE: receptor for advanced glycation end products
sRAGE: soluble RAGE
TGF: transforming growth factor
TJ: tight junction
TNF: tumor necrosis factor
ZO: zona occludens
